# The Melatonin Receptor Agonist Ramelteon Effectively Treats Insomnia and Behavioral Symptoms in Autistic Disorder

**DOI:** 10.1155/2014/561071

**Published:** 2014-05-14

**Authors:** Kentaro Kawabe, Fumie Horiuchi, Yasunori Oka, Shu-ichi Ueno

**Affiliations:** ^1^Department of Neuropsychiatry, Neuroscience, Ehime University Graduate School of Medicine, Shitsukawa, Toon, Ehime 791-0295, Japan; ^2^Center for Sleep Medicine, Ehime University Hospital, Shitsukawa, Toon, Ehime 791-0295, Japan

## Abstract

Children with autism spectrum disorders (ASD), including autistic disorder, frequently suffer from comorbid sleep problems. An altered melatonin rhythm is considered to underlie the impairment in sleep onset and maintenance in ASD. We report three cases with autistic disorder in whom nocturnal symptoms improved with ramelteon, a selective melatonin receptor agonist. Insomnia and behavior, assessed using the Clinical Global Impression-Improvement Scale, improved in two cases with 2 mg ramelteon and in the third case with 8 mg ramelteon. Our findings demonstrate that ramelteon is effective not only for insomnia, but for behavioral problems as well, in patients with autistic disorder.

## 1. Introduction

Autistic disorder is characterized by persistent deficits in social communication and social interaction and communication abilities and by the presence of restricted, repetitive patterns of behavior [1]. The category of autistic disorder is combined into autism spectrum disorders (ASD) in the Diagnostic and Statistical Manual of Mental Disorders, Fifth Edition (DSM-5) [2]. A systematic literature review found that autistic disorder in the DSM, Fourth Edition, Text Revision (DSM-IV-TR), is a fairly stable diagnosis supporting the more stringent DSM-5 criteria [3]. Many individuals with ASD present not only with diagnostic features but also with associated features, including intellectual impairment, motor deficits, and sleep problems. Sleep difficulties, particularly insomnia, occur in 50–80% of children with ASD [4, 5] and are often accompanied by child and family distress [6]. Sleep disturbance also exacerbates core and related symptoms of autism, including social interaction deficits, repetitive behaviors, affective problems, and hyperactivity/inattention [5, 7]. Therefore, interventions targeting sleep not only alleviate sleep difficulties, but also ameliorate core and related ASD symptoms and reduce familial distress.

Various neurobiological factors are known to modulate the sleep-wake cycle, and sleep problems are common in ASD. Neurotransmitter systems, such as gamma-aminobutyric acid (GABA), serotonin, and melatonin, are reported to be disturbed in ASD [8]. Melatonin is involved in promoting sleep onset and establishing a regular sleep-wake cycle. An altered melatonin rhythm seems to be responsible for the sleep onset and maintenance problems in ASD. Earlier studies showed that children with ASD have significantly lower mean concentrations of melatonin, mainly during the dark phase of the day, compared with controls [9]. Another study showed that melatonin may be helpful in treating sleep problems in children with ASD. In a randomized, placebo-controlled, double-blind, crossover study of 11 children with ASD, melatonin treatment effectively alleviated sleep difficulties [10]. In an open-label study of 107 children with ASD, 60% of the subjects treated with melatonin had an improvement in insomnia [11]. Furthermore, in an open-label dose-escalation study of 24 children with ASD conducted over a 14-week treatment period, melatonin improved symptoms after 1 week of supplementation, and the beneficial effects were maintained over several months. Melatonin effectively alleviated sleep and behavioral problems, reduced parenting stress, and was safe and well tolerated by the subjects [12].

Although melatonin is presently unavailable in many countries, including Japan, ramelteon, a melatonin agonist, is used for the treatment of insomnia in the United States and Asia. Ramelteon is a relatively new drug with high selectivity for the melatonin MT1 and MT2 receptors, which are located in the suprachiasmatic nucleus and have been implicated in the regulation of the sleep-wake cycle. Ramelteon has rapid oral absorption and a short elimination half-life [13] and has negligible affinity for a wide range of other binding sites in the central nervous system (including GABA, benzodiazepine, opioid, muscarinic, histamine, serotonin, and dopamine receptors) [14]. Here, we present three case reports of children with ASD treated with ramelteon.

## 2. Case Presentation

### 2.1. Case 1

**Figure 1:**
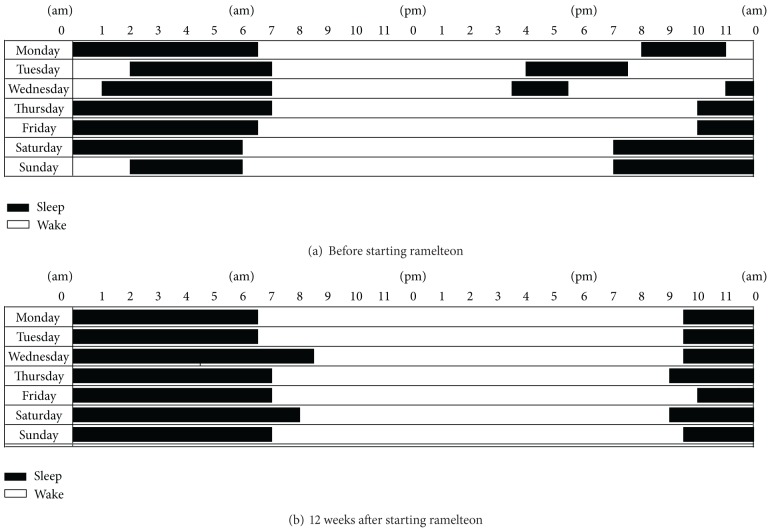
Sleep diaries of Case 1.

A 9-year-old boy was treated and followed since being diagnosed at the age of 4 years. He had a significant impairment in social skills, including reduced eye contact, lack of imaginative play, and absence of joint attention. He also had autistic regression. He had apparently normal language development when he aged 1 year; however, he lost acquired language abilities at the age of 2.5 years. He had a heavy acoustic hypersensitivity from childhood. He let out a strange noise and would panic, particularly on rainy days. He was diagnosed with autistic disorder according to the criteria in the DSM-IV-TR [2]. Background and clinical data are given in Table 1. At the age of 7 years, he had interfering behaviors consisting of hyperactivity, restless activities, and significant insomnia, including a delay in sleep onset of 2-3 h. Risperidone (0.5 mg/day) was started for sedating hyperactivity and restless activities. It was effective for approximately 1 year; however, restless activities and insomnia gradually relapsed. In addition, self-injury, such as knocking his head, appeared. Sodium valproate (200 mg/day) was added; however, he continued to exhibit self-injury. He could not keep regular sleep hours nor maintain a regular sleep-wake rhythm. As a result, he was not able to attend school. Ramelteon (2 mg/day) was started orally at 9:00  pm to advance the sleep phase, and other drugs were discontinued. Four weeks after starting ramelteon, he began to sleep before 11:00  pm and was able to maintain sleep throughout the night (Table 2). Adverse effects were not observed. He could wake up in a better mood in the morning than before. As he acquired the ability to maintain a regular sleep-wake rhythm, he was able to go outside and start attending school (Figure 1). His acoustic hypersensitivity became mildly improved and the frequency of panic attacks had been reduced two to three times weekly.

### 2.2. Case 2

An 11-year-old-boy was diagnosed with pervasive developmental disorders in another hospital at the age of 3 years. He was absent from most morning classes because he slept late at night and was unable to wake up until noon. His full intelligence quotient was 51, measured with the Wechsler Intelligence Scale for Children, Fourth Edition. He was diagnosed with autistic disorder and mental retardation according to criteria in the DSM-IV-TR. He was also observed to be hyperactive and impulsive. To treat the sleep-wake disturbance, ramelteon (2 mg/day) was administered at 10:00  pm. After 1 week, his sleep-wake phase had advanced and he was able to sleep from 12:00  pm to 9:00  am. He became able to keep regular sleep-wake rhythm both on weekdays and got up more comfortably. He started to go to school in the midmorning. In addition the symptom of hyperactivity at school and home was reduced. This beneficial effect of ramelteon has been maintained for over 50 weeks. No adverse effects, such as residual daytime sleepiness, were present during treatment with ramelteon.

### 2.3. Case 3

**Figure 2:**
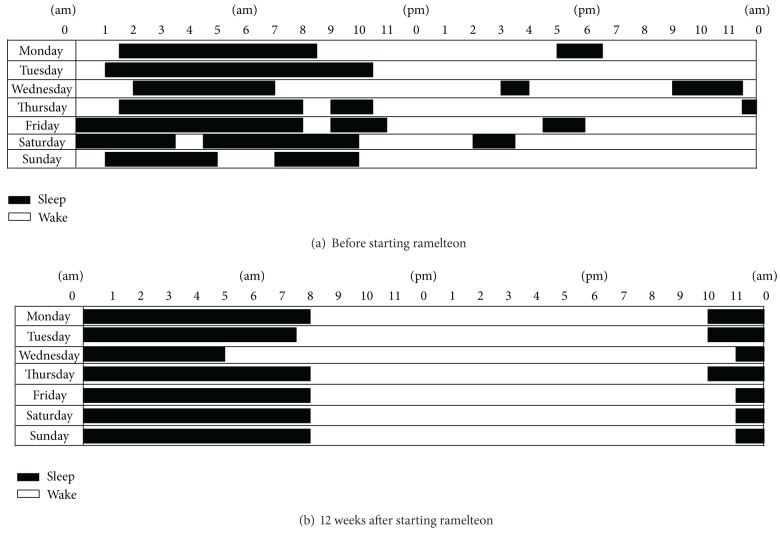
Sleep diaries of Case 3.

A 12-year-old-girl was followed since the age of 6 years. At the initial visit, she exhibited a delay in language development, hyperactivity, stereotyped movements, and perseverative behavior. She had autistic regression. She had apparently normal language development until she was aged 1 year but lost acquired language abilities at the age of 1.5 years. Her developmental quotient was 30. She was diagnosed with autistic disorder according to criteria in the DSM-IV-TR. When she was 9 years old, she suffered from an irregular sleep-wake pattern and midnight awakening with beating and kicking her mother. For sedation, risperidone (0.5 mg/day) was administered. Although her agitated mood improved, difficulty in initiating and maintaining sleep remained. Flunitrazepam (2 mg/day) and brotizolam (0.25 mg/day) were administered. However, her sleep disturbance did not improve. She suffered from partial convulsive seizures at the ages of 5, 10, and 13 years. Carbamazepine (100 mg/day) and lamotrigine (50 mg/day) were administered at the age of 12 years. She began to wake up at midnight, associated with repeated overeating, which caused a rapid weight gain. As sleep duration shortened to 6 h, from 10:00  pm to 4:00  am, ramelteon (4 mg/day) was administered orally at 9:00  pm. After 2 weeks, the dose was titrated to 8 mg/day. One week later, she was able to sleep from 10:00  pm to 6:00  am (Figure 2). The intermittent awakenings were reduced, and overeating at midnight disappeared although overeating before going to bed still continued. Ramelteon was effective for bedtime resistance for more than 1 year, and risperidone was discontinued.

## 3. Discussion

The three cases described here illustrate our clinical experience with ramelteon for children with autistic disorder. There are only few case reports on the use of ramelteon. Stigler et al. reported the potential effectiveness and tolerability of ramelteon for sleep disturbances in two males aged 7 and 18 with autistic disorder [15]. In our study, we presented not only male youth, but also female youth with autistic disorder, with sleep diaries for the evaluation of sleep-wake schedule.

Cases 1 and 2 demonstrate the effectiveness of 2 mg/day ramelteon. Case 3 required a relatively higher dose of the drug (8 mg/day). Ramelteon (4–32 mg/day) has been shown to work without dose dependency in both efficacy and adverse effects in randomized controlled trials [14, 16]. Our cases show that lower doses of ramelteon are effective for insomnia and behavioral symptoms in autistic disorder.

Cases 1 and 3 had a history of autistic regression. Giannotti et al. reported that a history of autistic regression is strongly associated with disrupted sleep [17]. The overall prevalence rate for autistic regression is 32.1% and occurs at a mean age of 1.78 years [18]. Over 50% of children with autism had at least one sleep problem during the second year of life, coinciding with the period during which autistic regression most frequently occurs. Several neurotransmitter systems, including GABA, serotonin, and melatonin, which have been implicated in promoting sleep and establishing a regular sleep-wake cycle, are affected in autism and may contribute to the sleep disruptions [19]. Abnormal melatonin regulation has been found in ASD, including elevated daytime melatonin and significantly decreased nocturnal melatonin. An abnormal circadian pattern may be due to dysfunction of the pineal gland in ASD [20], and perturbed synthesis and secretion of melatonin may increase the severity of ASD [21]. Furthermore, melatonin deficit is associated with social communication impairments [22]. Only a few clinical trials have examined the effects of melatonin on parameters other than sleep, such as autistic behavioral impairment. Improvement in communication [23], social withdrawal, and stereotyped behaviors [12] have been reported in children with ASD given melatonin treatment. In the present study, we found that ramelteon alleviates not only the sleep disturbance, but autistic behaviors as well.

There are several limitations to our report. We did not use instruments specialized for sleep evaluation, such as actigraphy or polysomnography. Consequently, the improvements in sleep are not definitive. In addition, we used the Clinical Global Impression-Improvement Scale for sleep and behavioral assessment. A more detailed evaluation is required to validate our behavioral findings. Furthermore, a larger sample size is needed to examine the efficacy of ramelteon in children with different autism subtypes.

In summary, ramelteon appears to be an effective treatment for sleep disorders as well as behavioral symptoms in autistic disorder during childhood. Further clinical studies are required to more thoroughly evaluate the effectiveness of ramelteon in children with ASD.

## Figures and Tables

**Table 1:** Patient background.

	Sex/age	Age diagnosed as ASD	CGI-S	School	Associated psychotropic medication (mg/day)
Case 1	M/9	4	6	Special school	Risperidone 0.5

Case 2	M/11	3	4	Special classroom	No drug

Case 3	F/12	6	6	Special school	Risperidone 0.5
					Brotizolam 0.25
					Flunitrazepam 2
					Carbamazepine 100
					Lamotrigine 50

The Clinical Global Impression-Severity (CGI-S) Scale is a 7-point scale used to assess symptom severity (1: normal, not ill; 2: minimally ill; 3: mildly ill; 4: moderately ill; 5: markedly ill; 6: severely ill; and 7: extremely ill) [24].

**Table 2:** Effectiveness of ramelteon.

	Ramelteon	Ramelteon (mg/day)		Effectiveness	
	Start age	Initial dose	Maintenance dose	CGI-I	Improvement
Case 1	9	2	2	2	Reduced LPS
					Reduced acoustic hypersensitivity
					Reduced panic attacks

Case 2	11	2	2	3	Reduced LPS
					Maintained sleep-wake rhythm
					Reduced hyperactivity

Case 3	12	4	8	2	Reduced LPS
					Increased TST
					Reduced overeating

The Clinical Global Impression-Improvement (CGI-I) Scale is used to assess the degree of symptom improvement or worsening (1: very much improved; 2: much improved; 3: moderately improved; 4: minimally improved; 5: no change; 6: minimally worse; 7: moderately worse; 8: much worse; and 9: very much worse). LPS: latency to persistent sleep; TST: total sleep time.
